# Metabolic and Hematological Consequences of Dietary Deoxynivalenol Interacting with Systemic *Escherichia coli* Lipopolysaccharide

**DOI:** 10.3390/toxins7114773

**Published:** 2015-11-16

**Authors:** Erik Bannert, Tanja Tesch, Jeannette Kluess, Jana Frahm, Susanne Kersten, Stefan Kahlert, Lydia Renner, Hermann-Josef Rothkötter, Sven Dänicke

**Affiliations:** 1 Institute of Animal Nutrition, Friedrich-Loeffler Institute (FLI), Federal Research Institute for Animal Health, Bundesallee 50, Braunschweig 38116, Germany; E-Mails: Erik.Bannert@fli.bund.de (E.B.); Tanja.Tesch@fli.bund.de (T.T.); Jana.Frahm@fli.bund.de (J.F.); Susanne.Kersten@fli.bund.de (S.K.); Sven.Dänicke@fli.bund.de (S.D.); 2 Institute of Anatomy, Otto-von-Guericke University Magdeburg, Leipziger Str. 44, Magdeburg 39120, Germany; E-Mails: stefan.kahlert@med.ovgu.de (S.K.); lydia.renner@med.ovgu.de (L.R.); hermann-josef.rothkoetter@med.ovgu.de (H.-J.R.)

**Keywords:** swine, deoxynivalenol, *E. coli* lipopolysaccharides, endotoxin, sepsis, blood gas, metabolism, glucose, inflammatory response

## Abstract

Previous studies have shown that chronic oral deoxynivalenol (DON) exposure modulated *Escherichia* *coli* lipopolysaccharide (LPS)-induced systemic inflammation, whereby the liver was suspected to play an important role. Thus, a total of 41 barrows was fed one of two maize-based diets, either a DON-diet (4.59 mg DON/kg feed, *n* = 19) or a control diet (CON, *n* = 22). Pigs were equipped with indwelling catheters for pre- or post-hepatic (portal *vs.* jugular catheter) infusion of either control (0.9% NaCl) or LPS (7.5 µg/kg BW) for 1h and frequent blood sampling. This design yielded six groups: CON_CON_jugular_-CON_portal_, CON_CON_jugular_-LPS_portal_, CON_LPS_jugular_-CON_portal_, DON_CON_jugular_-CON_portal_, DON_CON_jugular_-LPS_portal_ and DON_LPS_jugular_-CON_portal_. Blood samples were analyzed for blood gases, electrolytes, glucose, pH, lactate and red hemogram. The red hemogram and electrolytes were not affected by DON and LPS. DON-feeding solely decreased portal glucose uptake (*p* < 0.05). LPS-decreased partial oxygen pressure (*p*O_2_) overall (*p* < 0.05), but reduced *p*CO_2_ only in arterial blood, and DON had no effect on either. Irrespective of catheter localization, LPS decreased pH and base-excess (*p* < 0.01), but increased lactate and anion-gap (*p* < 0.01), indicating an emerging lactic acidosis. Lactic acidosis was more pronounced in the group DON_LPS_jugular_-CON_portal_ than in CON-fed counterparts (*p* < 0.05). DON-feeding aggravated the porcine acid-base balance in response to a subsequent immunostimulus dependent on its exposure site (pre- or post-hepatic).

## 1. Introduction

Due to its dependency on moderate climate conditions and its resistance to processing the *Fusarium* toxin, deoxynivalenol (DON) can be often found in toxicologically-relevant concentrations in cereals in temperate climate zones [1]. It is of special importance in pig production due to the high susceptibility of pigs, causing reduced feed intake and live weight gain, resulting in considerable economic losses [2,3,4].

Several studies indicate that DON influences the systemic inflammatory response. The toxin exerts immune modulatory effects on blood leukocytes depending on the dose and frequency of exposure. Different studies have shown that a low dose exposure to *Fusarium* toxins has an immune-stimulating effect due to an upregulation of transcriptional and post-transcriptional expression of cytokines, chemokines and inflammatory genes, whereas a high dose exposure has an immune-suppressive effect (reviewed in [5]). It has further been shown that exposure to DON causes an altered immune response [6,7,8] and liver cell metabolism [9,10] to a subsequent lipopolysaccharide (LPS) challenge *in vitro* and *in vivo*.

Lipopolysaccharides form the major component of the outer cell membrane of Gram-negative bacteria and are responsible for the onset of an inflammatory response in the case of systemic LPS entry [11]. Triggering similar (immune biological) pathways, a variety of infectious pathogens, such as Gram-positive and Gram-negative bacteria, viruses and fungi, leads to identical clinical sequelae commonly described with the term sepsis [12,13]. Since infections with Gram-negative bacteria contribute to a substantial part of the sepsis cases worldwide, LPS-induced systemic inflammation is a well-established sepsis model in animals and humans [11,14,15,16].

On a systemic level, the recognition of LPS by the immune system causes the release of pro-inflammatory cytokines. This leads to inflammation, apoptosis, causing endothelial dysfunction, and microcirculation thrombosis, resulting in perfusion heterogeneity and microcirculatory failure [12]. Clinically, these alterations manifest themselves in a variety of symptoms, such as hypothermia or hyperthermia, tachycardia, tachypnea, edema, central nervous dysfunction, leukocytosis and leukopenia [11,16,17]. In concurrence with a pronounced inflammatory response, blood analysis reveals often lactic acidosis [18,19,20], along with either dysglycemia depending on the stage of disease [17,18,21].

As DON can be ubiquitous in cereals and pigs might be sub-acutely exposed on the one hand, while LPS is always present in the environment and commensal intestinal microbiota, on the other hand, pigs might be often co-exposed to both toxins at the same time. The liver possesses a central role in LPS detoxification [22,23] or in metabolic and immunological homeostasis [24] of, and it has been shown before that chronic exposure to DON leads to altered liver cell metabolism [9,10]. We hypothesized that liver metabolism and hematological variables are altered in chronically-DON-fed animals during a subsequent LPS stimulus. An increase in systemic LPS can either be a consequence of a systemic infection [17] or an increased passage from portal-drained viscera [25]. In order to simulate these pathways of systemic LPS entrance and the consequences of a possible hepatic first-pass effect, we infused LPS pre- or post-hepatically. Arterial, jugular and portal blood metabolic variables were assessed to evaluate the role of the liver in this pathogenesis as a consequence of LPS infusion.

## 2. Results

### 2.1. Red Hemogram

Generally, neither DON nor LPS treatment influenced red hemogram variables irrespective of infusion site, and all values were always in their respective physiological range [26] (Table 1). Slight fluctuations combined with small variances in all measures contributed to significant time, catheter and group × catheter × time effects (Table 1).

**Table 1:** Effect of chronic enteral deoxynivalenol (DON) exposure and pre- or post-hepatic *E. coli* lipopolysaccharide (LPS) infusion on arterial, venous or portal red hemogram in pigs.

Parameter	Overall Mean	PSEM	*p*-Values			
			Group	Catheter	Time	G × C × T
RBC (×10 ^6^ cells/µL)	5.57	0.24	0.306	0.299	**<0.001**	0.467
Hct (%)	30.44	1.40	0.629	**0.006**	**<0.001**	0.415
Hgb (g/dL)	10.82	0.49	0.618	0.073 ^T^	**<0.001**	0.279
MCV (fL)	54.78	1.26	0.632	**<0.001**	0.981	**0.036**
MCH (g/dL)	19.48	0.52	0.602	0.144	**<0.001**	0.080 ^T^
MCHC (g/dL)	35.56	0.52	0.554	**0.001**	**<0.001**	0.429

Notes: RBC = red blood cells (reference: 5.5–8.5 × 10 ^6^ cells/µL ^1^); Hct = hematocrit (reference: 33%–45% ^1^); Hgb = hemoglobin (reference: 10.16–14.03 g/dL ^1^); MCV = mean corpuscular volume (reference: 50–65 fL ^1^); MCH = mean corpuscular hemoglobin (reference: 17–21 g/dL ^1^); MCHC = mean corpuscular hemoglobin concentration (reference: 30–35 g/dL ^1^); ^1^ = [26]; references in venous blood; ^T^ = trend (*p* ≤ 0.10); PSEM = pooled standard error of means. Barrows were either fed a DON contaminated ration (DON; 4.59 mg/kg feed) or control feed (CON) during 29 days. Infusion groups were divided as follows: pre-hepatic LPS infusion (CON_CON_jugular_-LPS_portal_, *n* = 7 and DON_CON_jugular_-LPS_portal_, *n* = 6), post-hepatic LPS infusion (CON_LPS_jugular_-CON_portal_, *n* = 8 and DON_LPS_jugular_-CON_portal_, *n* = 6), and control infusion (CON_CON_jugular_-CON_portal_, *n* = 7 and DON_CON_jugular_-CON_portal_, *n* = 7). Infusion from time 0 until 60 min with 7.5 µg LPS/kg BW in 0.9% saline. Feed was offered during 15 min prior to infusion start. Blood samples were collected at times: −30, 15, 30, 45, 60, 75, 90, 120, 150, 180 min.

**Figure 1:**
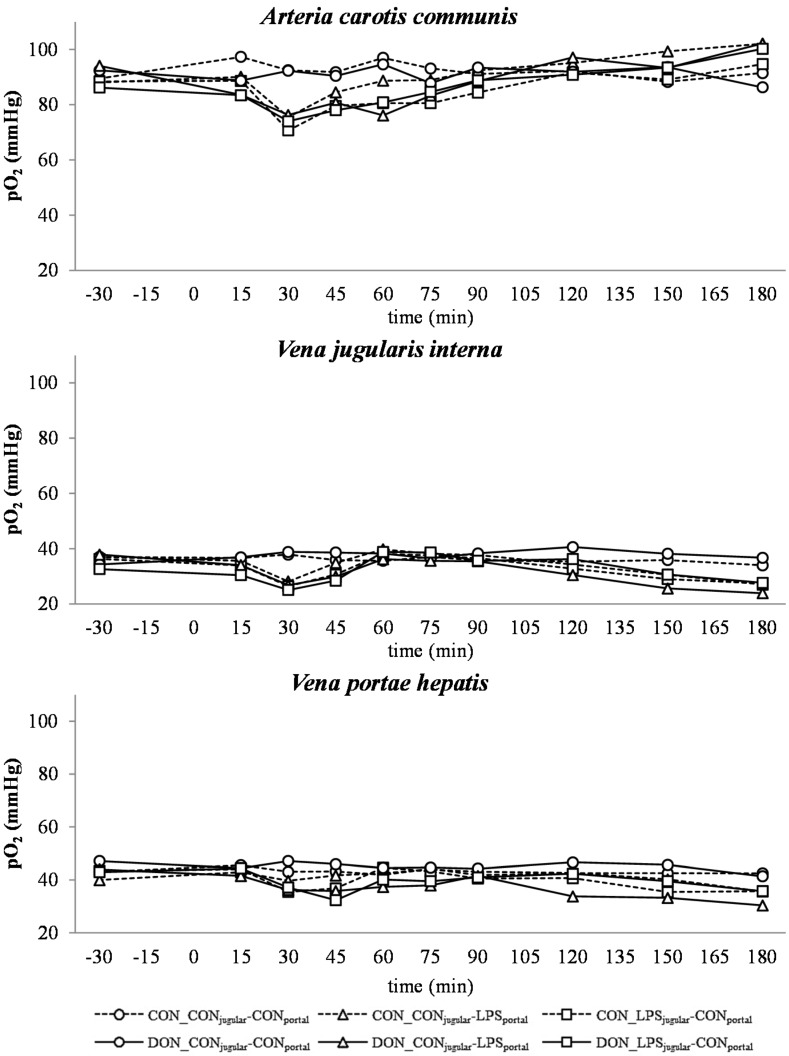
Effect of chronic enteral *Fusarium* toxin deoxynivalenol (DON) exposure and pre- or post-hepatic *E. coli* lipopolysaccharide (LPS) infusion on arterial, jugular or portal blood partial oxygen pressure (*p*O_2_) in pigs. Reference value: 98 mmHg in arterial blood [26]. Barrows were either fed a DON contaminated ration (4.59 mg/kg feed) or control feed during 29 days. Infusion groups were divided as follows: pre-hepatic LPS infusion (CON_CON_jugular_-LPS_portal_, *n* = 7 and DON_CON_jugular_-LPS_portal_, *n* = 6), post-hepatic LPS infusion (CON_LPS_jugular_-CON_portal_, *n* = 8 and DON_LPS_jugular_-CON_portal_, *n* = 6), and control infusion (CON_CON_jugular_-CON_portal_, *n* = 7 and DON_CON_jugular_-CON_portal_, *n* = 7). Infusion from time 0 until 60 min with 7.5 µg LPS/kg BW in 0.9% saline. Feed was offered during 15 min prior to infusion start. LSMeans. PSEM = 2.89. Significance: Group (G): *p* = 0.003; Catheter (C): *p* ≤ 0.001; Time (T): *p* ≤ 0.001; G × C × T: *p* ≤ 0.001.

### 2.2. Blood Gas Analysis

#### 2.2.1. Partial Oxygen Pressure

Arterial, jugular and portal partial oxygen pressures (*p*O_2_) are illustrated in Figure 1. On average, partial O_2_ pressures of the control group were 92.42 mmHg in arterial, 36.07 mmHg in jugular and 43.08 mmHg in portal blood (SEM = 1.21), respectively. The arterial *p*O2 was near the physiological reference value of 98 mmHg [26]. Between 15 and 60 min, a general decrease in *p*O_2_ in all LPS-infused groups (compared to their control groups) was observed at all infusion sites (*p* < 0.05), which started to return to base level at 60 min. The decrease was most pronounced in arterial blood. Thereafter, a subsequent increase in *p*O_2_ in all LPS-infused groups until 180 min was observed (*p* < 0.05). At jugular and portal sampling sites, *p*O_2_ decreased after 90 min, again below the control group level, and decreased thereafter until 120 min in jugular (*p* < 0.05) and 180 min in portal (*p* < 0.05) blood. A significant effect of DON exposure on arterial *p*O_2_ was observed at 60 min with pre-hepatic LPS-infused control-fed pigs starting to return to base level earlier than their DON-fed counter parts (*p* < 0.01). No effect of DON treatment was observed in post-hepatic infused animals. No DON effects were observed on jugular and portal *p*O_2_. Portal *p*O_2_ pressures were subjected more to fluctuations compared to arterial and portal *p*O_2_.

#### 2.2.2. Partial Carbon Dioxide Pressure

**Figure 2:**
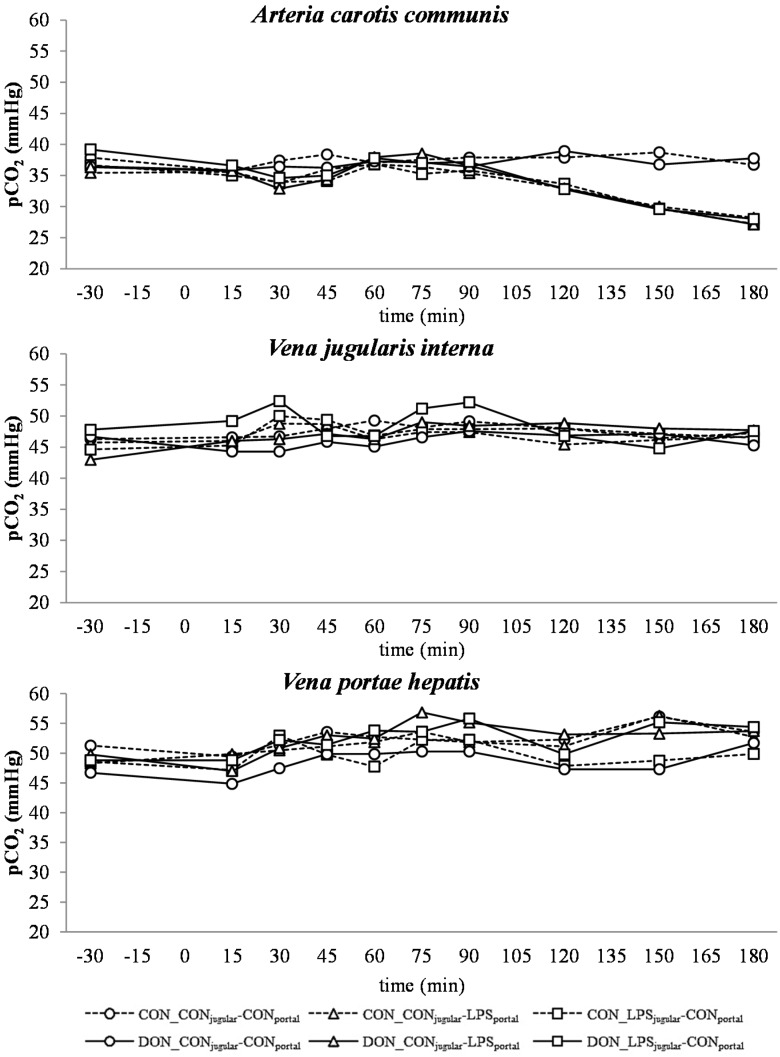
Effect of chronic enteral *Fusarium* toxin deoxynivalenol (DON) exposure and pre- or post-hepatic *E. coli* lipopolysaccharide (LPS) infusion on arterial, jugular or portal blood partial carbon dioxide pressure (*p*CO_2_) in pigs. Reference value: 50 mmHg in arterial blood [26]. Barrows were either fed a DON contaminated ration (4.59 mg/kg feed) or control feed during 29 days. Infusion groups were divided as follows: pre-hepatic LPS infusion (CON_CON_jugular_-LPS_portal_, *n* = 7 and DON_CON_jugular_-LPS_portal_, *n* = 6), post-hepatic LPS infusion (CON_LPS_jugular_-CON_portal_, *n* = 8 and DON_LPS_jugular_-CON_portal_, *n* = 6), and control infusion (CON_CON_jugular_-CON_portal_, *n* = 7 and DON_CON_jugular_-CON_portal_, *n* = 7). Infusion from time 0 until 60 min with 7.5 µg LPS/kg BW in 0.9% saline. Feed was offered during 15 min prior to infusion start. LSMeans. PSEM = 1.75. Significance: Group (G): *p* = 0.28; Catheter (C): *p* ≤ 0.001; Time (T): *p* ≤ 0.001; G × C × T: *p* ≤ 0.001.

Partial CO_2_ pressure (pCO_2_) of control group was 37.80 mmHg in arterial, 47.59 mmHg in jugular and 52.42 mmHg in portal blood (SEM = 0.75) on average. At the arterial and jugular sampling site, a pCO_2_ below the physiological reference range (50 mmHg) [26] was observed during the entire course of the trial and in all groups. Only arterial pCO_2_ was influenced by LPS treatment (Figure 2). From 120–180 min, a steady decrease was observed compared to the control group (*p* < 0.05). A slight portal pCO_2_ increase was observed from −30 min until 180 min in all groups (Figure 2). Chronic oral exposure to DON had no impact on pCO_2_ irrespective of LPS infusion site. In jugular, as well as portal blood samples, undirected fluctuations were observed.

### 2.3. Electrolytes

No significant effects of DON and LPS were observed on electrolytes (Na^+^, Cl^−^, K^+^, *i*Ca^2+^). However, catheter site and collecting time significantly influenced electrolyte concentrations (Table 2). Slight fluctuations were observed for Na^+^, K^+^ and *i*Ca^2+^ concentrations at different times, combined with minimal variation of the data, contributing to significant time and group × catheter × time effects. A significant catheter site effect was detected due to the generally higher jugular concentrations compared to arterial and portal levels at all groups. Electrolytes did not deviate from their respective physiological values [26,27] (Table 2).

**Table 2:** Effect of chronic enteral deoxynivalenol (DON) exposure and pre- or post-hepatic *E. coli* lipopolysaccharide (LPS) infusion on arterial, venous or portal blood electrolytes in pigs.

Parameter	Overall Mean	PSEM	*p*-Values			
			Group	Catheter	Time	G × C × T
Na^+^ (mmol/L)	142.90	1.15	0.449	**<0.001**	**<0.001**	0.629
Cl^−^ (mmol/L)	105.45	1.16	0.702	**<0.001**	**<0.001**	0.67
K^+^ (mmol/L)	4.28	0.16	0.585	**<0.001**	**<0.001**	**<0.001**
*i*Ca^2+^ (mmol/L)	1.37	0.03	0.985	**<0.001**	**<0.001**	**0.006**

Notes: Na^+^ = sodium (reference: 140–160 mmol/L^1^); Cl^−^ = chloride (reference: 102–106 mmol/L^1^); K^+^ **=** potassium (reference: 4.0–5.0 mmol/L ^1^); *i*Ca^2+^ = calcium iones (reference: 0.87–1.45 mmol/L ^2^); ^1^ = [26]; ^2^ = [27]; references in venous blood; ^T^ = trend (*p *≤ 0.10); PSEM = pooled standard error of Means. Barrows were either fed a DON contaminated ration (DON; 4.59 mg/kg feed) or control feed (CON) during 29 days. Infusion groups were divided as follows: pre-hepatic LPS infusion (CON_CON_jugular_-LPS_portal_, *n* = 7 and DON_CON_jugular_-LPS_portal_, *n* = 6), post-hepatic LPS infusion (CON_LPS_jugular_-CON_portal_, *n* = 8 and DON_LPS_jugular_-CON_portal_, *n* = 6), and control infusion (CON_CON_jugular_-CON_portal_, *n* = 7 and DON_CON_jugular_-CON_portal_, *n* = 7). Infusion from time 0 until 60 min with 7.5 µg LPS/kg BW in 0.9% saline. Feed was offered during 15 min prior to infusion start. Blood samples were collected at times: −30, 15, 30, 45, 60, 75, 90, 120, 150, 180 min.

### 2.4. Glucose

**Figure 3:**
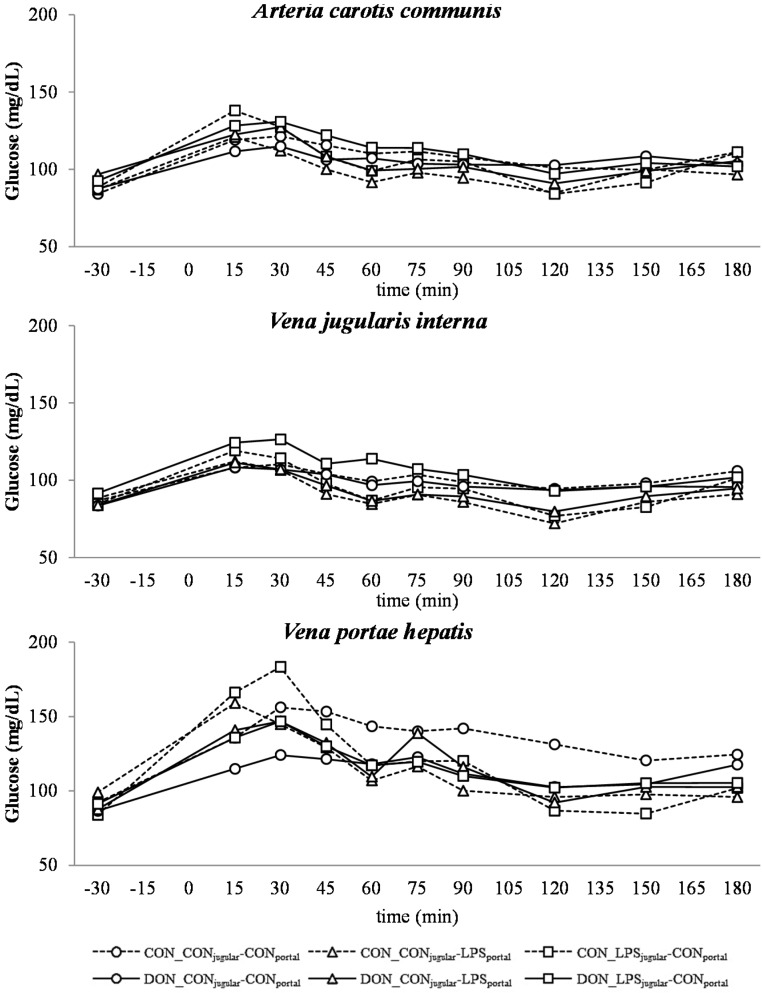
Effect of chronic enteral *Fusarium* toxin deoxynivalenol (DON) exposure and pre- or post-hepatic *E. coli* lipopolysaccharide (LPS) infusion on arterial, jugular or portal blood glucose in pigs. Reference value: 70–115 mg/dL in venous blood [26]. Barrows were either fed a DON contaminated ration (4.59 mg/kg feed) or control feed during 29 days. Infusion groups were divided as follows: pre-hepatic LPS infusion (CON_CON_jugular_-LPS_portal_, *n* = 7 and DON_CON_jugular_-LPS_portal_, *n* = 6), post-hepatic LPS infusion (CON_LPS_jugular_-CON_portal_, *n* = 8 and DON_LPS_jugular_-CON_portal_, *n* = 6), and control infusion (CON_CON_jugular_-CON_portal_, *n* = 7 and DON_CON_jugular_-CON_portal_, *n* = 7). Infusion from time 0 until 60 min with 7.5 µg LPS/kg BW in 0.9% saline. Feed was offered during 15 min prior to infusion start. LSMeans. PSEM = 1.75. Significance: Group (G): *p* = 0.28; Catheter (C): *p* ≤ 0.001; Time (T): *p* ≤ 0.001; G × C × T: *p* ≤ 0.001. Table illustrates differences between DON- and CON-fed control-infused groups at different times. ^T^ = trend (*p* ≤ 0.10).

A post-prandial increase in glucose until 15–30 min and a subsequent decrease until time 120 min to the base level was observed in all groups (Figure 3), most pronounced at the portal sampling site. The control (CON)-fed animals generally exhibited higher glucose levels at the portal sampling site at 30–45 min (depending on group) than DON-fed animals (Figure 3). The control group (CON_CON_jugular_-CON_portal_) maintained elevated post-prandial glucose levels for nearly the entire time course (significantly higher than other groups at 60 min, 90 min and 120 min; *p* < 0.05 at 120 min).

**Table:** 

*post hoc* test (*p*-value) CON_CON-CON *vs*. DON_CON-CON										
**time (min)**	−30	15	30	45	60	75	90	120	150	180
***A.*** ***carotis***	0.819	0.599	0.652	0.518	0.833	0.587	0.745	0.912	0.546	0.587
***V.*** ***jugularis***	0.812	0.991	0.811	0.983	0.865	0.756	0.836	0.950	0.885	0.465
***V.*** ***portae***	0.686	0.131	0.024	0.021	0.066^T^	0.207	0.028	0.043	0.247	0.632

**Figure 4:**
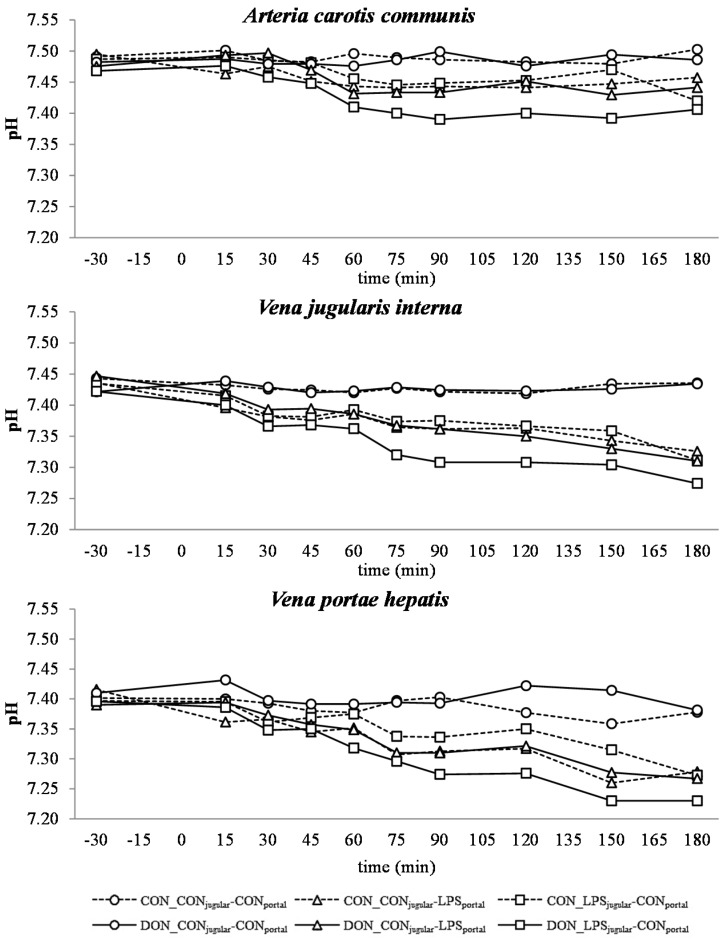
Effect of chronic enteral Fusarium toxin deoxynivalenol (DON) exposure and pre- or post-hepatic *E. coli* lipopolysaccharide (LPS) infusion on arterial, jugular or portal blood pH in pigs. Reference value: 7.42 in arterial blood [26]. Barrows were either fed a DON contaminated ration (4.59 mg/kg feed) or control feed during 29 days. Infusion groups were divided as follows: pre-hepatic LPS infusion (CON_CON_jugular_-LPS_portal_, *n* = 7 and DON_CON_jugular_-LPS_portal_, *n* = 6), post-hepatic LPS infusion (CON_LPS_jugular_-CON_portal_, *n* = 8 and DON_LPS_jugular_-CON_portal_, *n* = 6), and control infusion (CON_CON_jugular_-CON_portal_, *n* = 7 and DON_CON_jugular_-CON_portal_, *n* = 7). Infusion from time 0 until 60 min with 7.5 µg LPS/kg BW in 0.9% saline. Feed was offered during 15 min prior to infusion start. LSMeans. PSEM = 0.02. Significance: Group (G): *p* ≤ 0.001; Catheter (C): *p* ≤ 0.001; Time (T): *p* ≤ 0.001; G × C × T: *p* ≤ 0.001. Table illustrates differences between DON and CON fed post-hepatic LPS infused groups at different times. ^T^ = trend (*p* ≤ 0.10).

**Table:** 

*post hoc* test (p-value) CON_LPS-CON vs. DON_LPS-CON										
**time (min)**	−30	15	30	45	60	75	90	120	150	180
***A.*** ***carotis***	0.499	0.611	0.327	0.236	0.105	0.105	0.038	0.061 ^T^	0.006	0.623
***V.*** ***jugularis***	0.636	0.585	0.548	0.630	0.268	0.051 ^T^	0.015	0.035	0.047	0.176
***V.*** ***portae***	0.975	0.736	0.637	0.495	0.039	0.132	0.024	0.007	0.002	0.129

**Figure 5:**
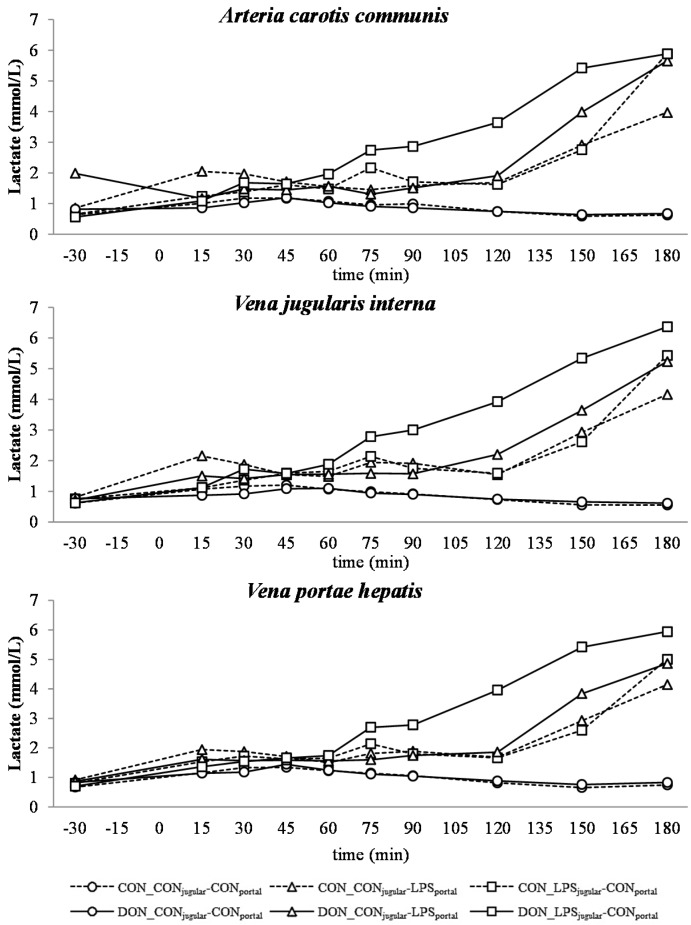
Effect of chronic enteral *Fusarium* toxin deoxynivalenol (DON) exposure and pre- or post-hepatic *E. coli* lipopolysaccharide (LPS) infusion on arterial, jugular or portal blood lactate in pigs. Reference: 0.84 ± 0.24 mmol/L in venous blood [28]. Barrows were either fed a DON contaminated ration (4.59 mg/kg feed) or control feed during 29 days. Infusion groups were divided as follows: pre-hepatic LPS infusion (CON_CON_jugular_-LPS_portal_, *n* = 7 and DON_CON_jugular_-LPS_portal_, *n* = 6), post-hepatic LPS infusion (CON_LPS_jugular_-CON_portal_, *n* = 8 and DON_LPS_jugular_-CON_portal_, *n* = 6), and control infusion (CON_CON_jugular_-CON_portal_, *n* = 7 and DON_CON_jugular_-CON_portal_, *n* = 7). Infusion from time 0 until 60 min with 7.5 µg LPS/kg BW in 0.9% saline. Feed was offered during 15 min prior to infusion start. LSMeans. PSEM = 0.56. Significance: Group (G): *p* ≤ 0.001; Catheter (C): *p* = 0.78; Time (T): *p* ≤ 0.001; G × C × T: *p* ≤ 0.001. Table illustrates differences between DON and CON fed post-hepatic LPS infused groups at different times. ^T^ = trend (*p* ≤ 0.10).

**Table:** 

*post hoc* test (p-value) CON_LPS-CON *vs*. DON_LPS-CON										
**time (min)**	−30	15	30	45	60	75	90	120	150	180
***A.carotis***	0.912	0.845	0.714	0.969	0.558	0.484	0.161	0.014	0.001	0.999
***V.jugularis***	0.871	0.993	0.656	0.993	0.775	0.424	0.120	0.004	0.001	0.245
***V.portae***	0.904	0.838	0.824	0.965	0.911	0.484	0.223	0.004	<0.001	0.252

**Figure 6:**
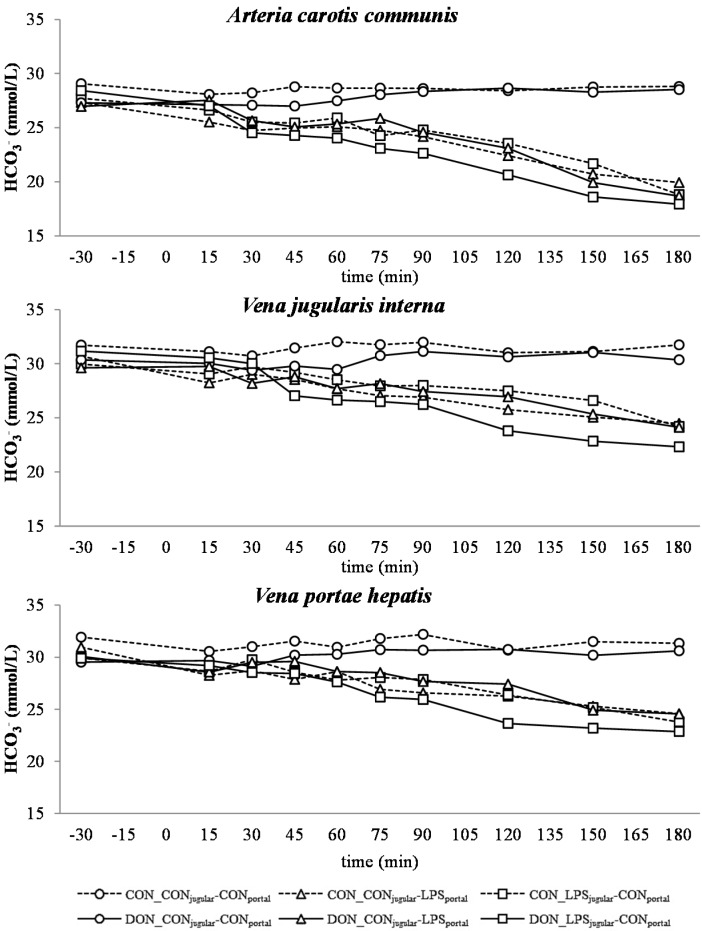
Effect of chronic enteral *Fusarium* toxin deoxynivalenol (DON) exposure and pre- or post-hepatic *E. coli* lipopolysaccharide (LPS) infusion on arterial, jugular or portal blood bicarbonate (HCO_3_^−^) in pigs. Reference range: 20–30 mmol/L in arterial blood [26]. Barrows were either fed a DON contaminated ration (4.59 mg/kg feed) or control feed during 29 days. Infusion groups were divided as follows: pre-hepatic LPS infusion (CON_CON_jugular_-LPS_portal_, *n* = 7 and DON_CON_jugular_-LPS_portal_, *n* = 6), post-hepatic LPS infusion (CON_LPS_jugular_-CON_portal_, *n* = 8 and DON_LPS_jugular_-CON_portal_, *n* = 6), and control infusion (CON_CON_jugular_-CON_portal_, *n* = 7 and DON_CON_jugular_-CON_portal_, *n* = 7). Infusion from time 0 until 60 min with 7.5 µg LPS/kg BW in 0.9% saline. Feed was offered during 15 min prior to infusion start. LSMeans. PSEM = 1.18. Significance: Group (G): *p* ≤ 0.001; Catheter (C): *p* ≤ 0.001; Time (T): *p* ≤ 0.001; G × C × T: *p* ≤ 0.001. Table illustrates differences between DON and CON fed post-hepatic LPS infused groups at different times,^T^ = trend (*p* ≤ 0.10).

**Table:** 

*post hoc* test (p-value) CON_LPS-CON *vs*. DON_LPS-CON										
**time (min)**	−30	15	30	45	60	75	90	120	150	180
***A.carotis***	0.639	0.789	0.506	0.443	0.215	0.431	0.153	0.054 ^T^	0.042	0.558
***V.jugularis***	0.423	0.314	0.835	0.145	0.206	0.327	0.238	0.014	0.012	0.205
***V.portae***	0.980	0.714	0.408	0.934	0.896	0.202	0.195	0.066 ^T^	0.181	0.539

**Figure 7:**
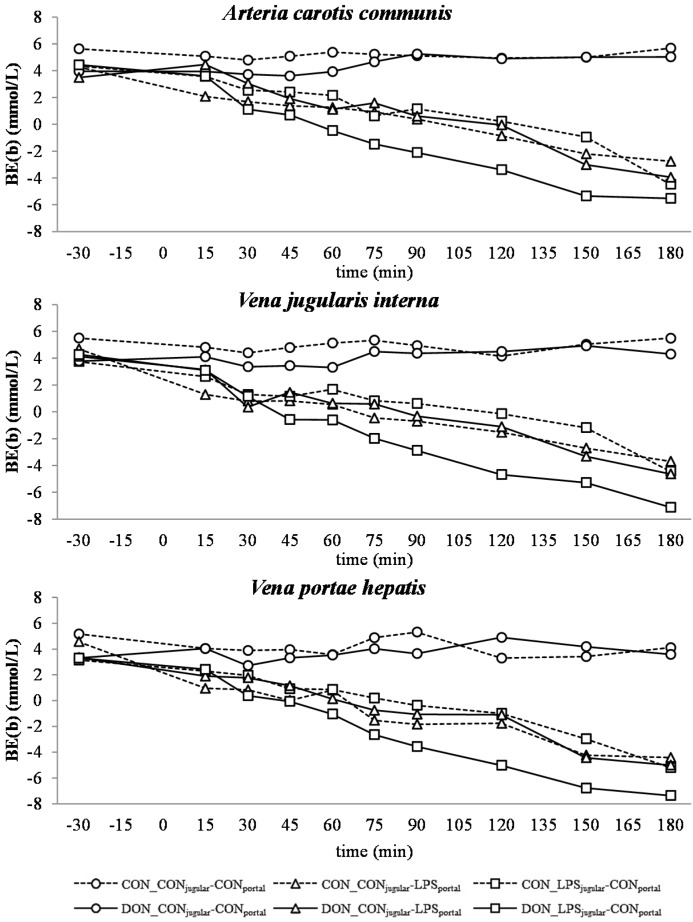
Effect of chronic enteral *Fusarium* toxin deoxynivalenol (DON) exposure and pre- or post-hepatic *E. coli* lipopolysaccharide (LPS) infusion on arterial, jugular or portal blood base-excess (BE(b)) in pigs. Reference range: −3.5–3.5 mmol/L in arterial blood [26]. Barrows were either fed a DON contaminated ration (4.59 mg/kg feed) or control feed during 29 days. Infusion groups were divided as follows: pre-hepatic LPS infusion (CON_CON_jugular_-LPS_portal_, *n* = 7 and DON_CON_jugular_-LPS_portal_, *n* = 6), post-hepatic LPS infusion (CON_LPS_jugular_-CON_portal_, *n* = 8 and DON_LPS_jugular_-CON_portal_, *n* = 6), and control infusion (CON_CON_jugular_-CON_portal_, *n* = 7 and DON_CON_jugular_-CON_portal_, *n* = 7). Infusion from time 0 until 60 min with 7.5 µg LPS/kg BW in 0.9% saline. Feed was offered during 15 min prior to infusion start. LSMeans. PSEM = 1.18. Significance: Group (G): *p* ≤ 0.001; Catheter (C): *p* ≤ 0.001; Time (T): *p* ≤ 0.001; G × C × T: *p* ≤ 0.001. Table illustrates differences between DON and CON fed post-hepatic LPS infused groups at different times ^T^ = trend (*p* ≤ 0.10).

**Table:** 

*post hoc *test (p-value) CON_LPS-CON *vs*. DON_LPS-CON										
**time (min)**	−30	15	30	45	60	75	90	120	150	180
***A.carotis***	0.957	0.988	0.413	0.324	0.133	0.226	0.062 ^T^	0.039	0.012	0.545
***V.jugularis***	0.757	0.787	0.935	0.310	0.186	0.103	0.042	0.009	0.018	0.119
***V.portae***	0.931	0.941	0.364	0.566	0.268	0.099^T^	0.064 ^T^	0.020	0.027	0.214

**Figure 8:**
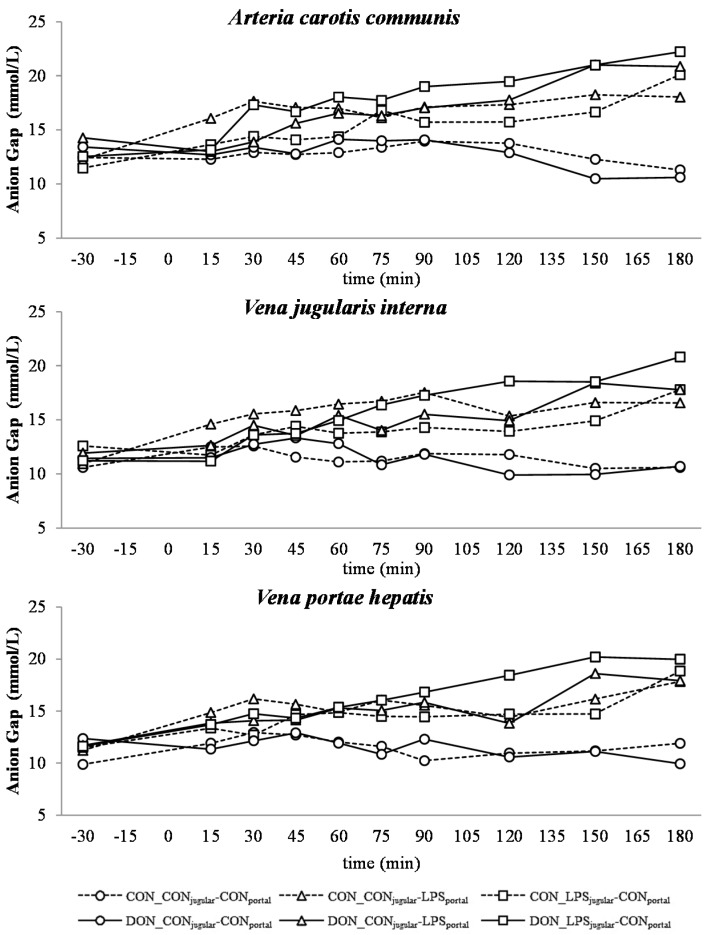
Effect of chronic enteral *Fusarium* toxin deoxynivalenol (DON) exposure and pre- or post-hepatic *E. coli* lipopolysaccharide (LPS) infusion on arterial, jugular or portal blood anion-gap Gap (K^+^) in pigs. Reference range: 10–25 mmol/L in venous blood [27]. Barrows were either fed a DON contaminated ration (4.59 mg/kg feed) or control feed during 29 days. Infusion groups were divided as follows: pre-hepatic LPS infusion (CON_CON_jugular_-LPS_portal_, *n* = 7 and DON_CON_jugular_-LPS_portal_, *n* = 6), post-hepatic LPS infusion (CON_LPS_jugular_-CON_portal_, *n* = 8 and DON_LPS_jugular_-CON_portal_, *n* = 6), and control infusion (CON_CON_jugular_-CON_portal_, *n* = 7 and DON_CON_jugular_-CON_portal_, *n* = 7). Infusion from time 0 until 60 min with 7.5 µg LPS/kg BW in 0.9% saline. Feed was offered during 15 min prior to infusion start. LSMeans. PSEM = 1.22. Significance: Group (G): *p* ≤ 0.001; Catheter (C): *p* ≤ 0.001; Time (T): *p* ≤ 0.001; G × C × T: *p* ≤ 0.001. Table illustrates differences between DON and CON fed post-hepatic LPS infused groups at different times.^T^ = trend (*p* ≤ 0.10).

**Table:** 

*post hoc *test (*p*-value) CON_LPS-CON *vs.* DON_LPS-CON										
**time (min)**	−30	15	30	45	60	75	90	120	150	180
***A.carotis***	0.564	0.792	0.115	0.162	0.048	0.612	0.075 ^T^	0.043	0.019	0.253
***V.jugularis***	0.451	0.759	1.000	0.716	0.516	0.170	0.102	0.011	0.048	0.095 ^T^
***V.portae***	0.951	0.855	0.268	0.850	0.784	0.392	0.192	0.041	0.003	0.535

### 2.5. Acid-Base Balance

In all LPS-infused pigs, lactic acidosis was induced, and the acid-base balance variables were altered accordingly (Figure 4, Figure 5, Figure 6, Figure 7 and Figure 8). Compared to the control group (CON_CON_jugular_-CON_portal_), pH (Figure 4), bicarbonate (Figure 6) and base excess (BE; Figure 7) decreased and lactate (Figure 5) and anion-gap (AG; Figure 8) increased significantly (*p* < 0.05). The control group stayed in the physiological range [26] for pH (arterial), lactate (venous) [28], HCO_3_^−^ (arterial) and AG (venous) [27], but BE (arterial) was above the reference range over the course of the trial with individual variations (5.23 mmol/L ± 0.79; mean ± SE).

DON treatment had no effect on control and pre-hepatic LPS-infused groups, but a significant DON effect was observed in post-hepatic LPS-infused animals. Lactic acidosis was most pronounced in post-hepatic LPS-infused DON-fed pigs (DON_LPS_jugular_-CON_portal_) at different times in comparison to their control-fed counterparts (CON_LPS_jugular_-CON_portal_) and the pre-hepatic LPS-infused groups (*p* < 0.05) (shown in the *post hoc* tables, Figure 4, Figure 5, Figure 6, Figure 7 and Figure 8). The DON_LPS_jugular_-CON_portal_ group had a different time course compared to the other LPS groups for all variables of acid-base balance. This was most apparent in lactate, with a significant increase compared to the control already at 75–180 min at all catheters in contrast to the other LPS groups, which were only significantly different from the control at 150 min and 180 min. Additionally, we observed a chronological sequence of lactic acidosis variables compared to the control: *p*O_2_, HCO_3_^−^ and BE already changed at 30–45 min, followed by pH at 60 min, and as the last variable, lactate rose significantly at 75 min.

## 3. Discussion

In this study, LPS was used to induce an inflammatory response. All LPS-treated pigs exhibited typical clinical symptoms of an acute phase response [16,29,30], such as an increased respiratory rate, fever, tremor, cyanosis, followed by hyperemic conjunctivae, injected episcleral vessels or leukopenia (Tesch *et al.*, 2015, submitted, [31]). In all groups at time −30 min and within the control group over the course of the trial, no significant alterations of metabolic and hematological variables were observed, and all parameters were within their physiological range. It therefore can be stated that the performed manipulations, such as the surgery and the sampling procedure, did not constitute confounding factors.

All LPS-treated animals exhibited a lactic acidosis as a consequence of LPS infusion [13]. Taking into consideration the observed *p*O_2_, *p*CO_2_ and lactate concentrations, we deduced that at first, acidosis was caused by a decreased *p*O_2_ (respiratory acidosis) and later on originated from an increase in systemic lactate concentration (metabolic acidosis) [32]. This is also mirrored in the variables BE and AG, which are a reflection of the different variables that affect the acid-base balance [33]. The alterations observed in AG can solely be ascribed to changes in HCO_3_^−^ concentrations, since other electrolytes (Na^+^, K^+^, Cl^−^, Ca^2+^) were not influenced by the LPS challenge.

Since erythrocytes occupy a central role in oxygen transport, metabolism, as well as the acid-base balance, we also assessed red hemogram variables. No biologically-relevant influence of DON or LPS was observed on red hemogram variables at any time during the trial. This is in line with a study of Grenier *et al.* [34] in which the effects of the mycotoxins DON and fumonisin, alone or in combination with a subcutaneous ovalbumin injection on different hematology variables, were investigated. These results are further confirmed by two recent studies [35,36] investigating the influence of low-dose (≤2 mg/kg feed) chronic oral DON exposure on red hemogram variables and electrolytes in piglets and pre-puberal gilts, respectively. However, in both studies, neither biologically-relevant alterations in red hemogram nor blood electrolytes were observed after four weeks of chronic oral DON exposure. Few studies have investigated the influence of an acute inflammatory response on sodium-potassium transport in red blood cells and skeletal muscle. Suri and colleagues [37] and Illner *et al.* [38] observed hyponatremia and hyperpotassemia and attributed this to alteration in the transport capacity of the RBC Na^+^/K^+^ pump. This change in ion transport across RBC and skeletal muscles has also been observed in other studies [39,40], but this hypothesis was challenged later on [41,42]. In our study, we did not detect any changes in blood electrolytes and, based on these previous articles, might speculate that the sodium-potassium transport in red blood cells and skeletal muscle after an LPS-challenge was not changed.

A decrease in systemic *p*O_2_ during the initial state of an acute inflammatory response has been documented before and is probably caused by a decreased cardiac output, as well as alterations in the respiratory rate and depth [43]. This assumption is supported by the concurrent observed increase in respiratory rate in LPS-infused animals during this trial (Tesch *et al.*, 2015, submitted, [31]). After an initial decrease of arterial *p*O_2_, a continuous increase during the rest of the trial, accompanied by a decrease in jugular *p*O_2_ and no alterations in jugular pCO_2_, was observed. This observation is indicative of an increase in tissue oxygen consumption, rather than a decrease in overall oxygen availability. These findings are in line with previous studies showing that the hyperlactatemia observed during sepsis is most likely caused by alterations in the glycolytic pathway, rather than hypoxia, along with increased tissue oxygen consumption [44]. In human studies, Revelly and co-workers [45] described an increased glucose and lactate rate of appearance in the blood of septic patients compared to healthy subjects. Furthermore, the hyperlactatemia resulted from an increased endogenous lactate production in sepsis, whereas lactate clearance was not altered compared to healthy patients, confirming the impact of altered glycolytic pathways in the development of lactic acidosis. It is further assumed that during a state of acute inflammatory response, the rate of pyruvate formation exceeds the oxidative capacity of mitochondria, causing an accumulation of pyruvate, and thereby, an increase in lactate formation [46]. This is further potentiated by a decrease in lactate utilization [47]. Besides an alteration in the glycolytic pathway, tissue perfusion heterogeneity is being put forward as a possible reason for hyperlactatemia accompanied by physiological systemic *p*O_2_ during sepsis by Gutierrez and colleagues [18]. Under shock conditions, the blood circulation is centralized to vital organs, and non-vital tissues are characterized by a compromised peripheral vascular perfusion [48]. In our study, we clinically observed cyanosis of the extremities and dermographism in five out of 28 LPS-treated pigs (Tesch *et al.*, 2015, submitted, [31]), as well as liver hemorrhage in LPS-treated animals (Renner *et al.*, 2015, [49]). In contrast to our observations, other studies with a similar setup reported dermographism in most of the LPS-treated animals, as well as macroscopic and microscopic intrahepatic hyperperfusion [8]. These symptoms can be attributed, amongst others, to tissue perfusion heterogeneity and, thus, would fit the hypothesis voiced earlier [18]. Further, an increased lactic acid output of leukocytes due to an increased glycolysis has been suggested as a contributing factor to the lactic acidosis observed in septic animals [50]. In our study, leukocytes in particular were severely affected by LPS and an enhanced glycolysis, and thus, the output of lactic acid into the blood might have contributed to the present lactic acidosis (Tesch *et al.*, 2015, submitted, [31]).

There was no difference between pre- or post-hepatic LPS infusion in CON-fed animals concerning the acid-base balance. An influence of DON was only observed in post-hepatic LPS-infused animals, whereas no dietary impact in pre-hepatic LPS-infused animals was observed regarding different acid-base balance variables in our trial. These results suggest a DON-related priming of post-hepatic cells involved in the exacerbation of metabolic disorders caused by LPS stimulation. Moreover, the lack of an assumed partial hepatic LPS clearance in post-hepatic LPS-infused DON-fed pigs compared to their pre-hepatic LPS-infused DON-fed counterparts might have triggered latent interactions between LPS and DON. Previously, it has been shown that an exposure to DON causes an altered immune response due to an upregulation of the transcriptional and post-transcriptional expression of cytokines, chemokines and inflammatory genes in porcine *in vitro* and *in vivo* studies [6,7,8]. In rodent studies, LPS priming of animals prior to DON exposure resulted in a stronger cytokine response compared to vehicle-treated animals [51], as well as simultaneous LPS and DON treatment of RAW264.7 macrophages [52]. However, we observed a uniform increase of TNF-α after 30 min with peak values at 60 min in all LPS-infused pigs, irrespective of the site of infusion or dietary treatment. Therefore, in our study, we could not confirm a superinduction of TNF-α in LPS-treated animals fed with a DON-contaminated diet (Tesch *et al.*, 2015, submitted, [31]).

Similar to other studies that have investigated the influence of a systemic inflammatory response on blood glucose levels, we observed an initial hyperglycemia followed by eu- and hypoglycemia [21,53]. However, this kinetic was not significantly distinguishable from the post-prandial increase in blood glucose, which was observed in all groups as LPS infusion superimposed with the post-prandial effects.

The DON fed animals exhibited a markedly lower portal glucose level, which can most likely be attributed to the negative effects of DON on glucose transport across the intestinal barrier, as described previously by Halawa *et al.* [54]. This effect was also observed in several other studies using different animal and *in vitro* models. In chickens, DON inhibited the jejunal SGLT-1 activity (sodium-linked glucose transporter 1), responsible for active glucose uptake into enterocytes from lumen [55]. Furthermore, in human cell line HT-29, SGLT-1, GLUT-5 (glucose transporter 5, d-fructose associated) and GLUT-1 (passive d-glucose transporter) were inhibited by DON in a dose-dependent manner [56]. However, other studies in swine did not confirm this effect on SGLT-1 activity in brush border vesicles derived from the jejunum [57]. In addition to this DON effect, an additive effect of LPS infusion was detected. All LPS-infused groups had lower portal glucose concentrations 45 min after infusion start compared to the total control group. Generally under shock conditions, hypoperfusion of the intestinal tract can be observed [58,59], and therefore, it can be hypothesized that the transport capacity of the intestinal mucosa is impaired. Furthermore, in several LPS studies, an inhibitory effect on intestinal glucose transport was observed, for instance decreased GLUT-5 [60] and SGLT-1 [61] levels in LPS-treated rabbits. Furthermore, Amador and co-authors [62] observed an inhibitory effect of TNF-α on SGLT-1 *ex vivo* in rabbit’s intestine. We could thus hypothesize that in our study, the impaired portal glucose uptake in LPS-treated animals (45 min–180 min) might be, at least partially, attributed to an impairment in SGLT-1 transport capacity due to the increase in TNF-α (Tesch *et al.*, 2015, submitted, [31]).

Our data suggest that chronic oral exposure to DON exacerbates lactic acidosis by a post-hepatic LPS-induced systemic inflammation, while a pre-hepatic LPS stimulation did not result in such amplification. This different responsiveness between pre- and post-hepatic-infused animals was not observed within the control-fed groups.

## 4. Experimental Section

Animal experiments were conducted according to the EC regulations concerning the protection of experimental animals and the guidelines of the German Animal Welfare Act approved by the Lower Saxony State Office for Consumer Protection and Food Safety (Lower Saxony State Office for Consumer Protection and Food Safety; File Number 33.4-42502-04-13/1274).

### 4.1. Experimental Design and Procedures

**Figure 9:**
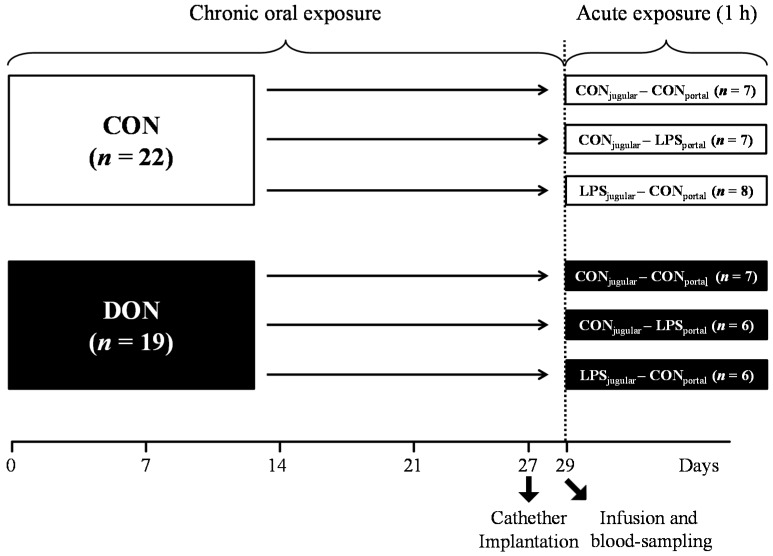
Experimental design.

A total of 41 barrows (German landrace, Mariensee, Germany) were randomly assigned to either a group receiving natural DON-contaminated feed (DON; 4.59 mg DON/kg feed; *n* = 19) or a control group (CON; *n* = 22) control diet (Table 3). Experimental groups, their treatment and the number of animals are illustrated in Figure 9. The pigs had an average initial weight of 25.8 ± 3.7 kg (means ± SD) and were fed restrictively with 2 single portions of 700 g per day, mixed with water and provided as mash. All barrows were housed in individual floor pens during the first 21 days of the trial and subsequently transferred into individual metabolism crates (described in [63]) until Day 29.

**Table 3:** Diet composition, based on air dry matter (ADM) = 88.37%.

Ingredients	CON %	DON %
barley	53.30	53.30
maize (non contaminated)	15.00	7.50
maize (contaminated)	-	7.50
soybean meal	20.00	20.00
rapeseed	5.00	5.00
soybean oil	2.00	2.00
Premix ^1^	3.00	3.00
Lysine-HCl	0.40	0.40
l-Threonine	0.12	0.12
dl-Methionine	0.15	0.15
HCl-insoluble ash ^2^	1.00	1.00
analysed composition	g/kg ADM	g/kg ADM
crude protein	196.85	194.83
crude fat	47.48	46.51
crude ash	69.70	69.51
crude fiber	51.26	49.32
deoxynivalenol mg/kg	0.12	4.59

^1^ Provided per kilogram of premix: Ca 25 g, P 6 g, Na 5.5 g, Mg 1 g, Fe 4000 mg, Cu 500 mg, Mn 2670 mg, Zn 3340 mg, I 67 mg, Se 13.5 mg, Co 8.3 mg, bas. Co-II-carb-monohydrat 8.3 mg, vitamin A 400000 I.U., vitamin D3 40000 I.U., vitamin E 1200 mg, vitamin B_1_ 37.5 mg, vitamin B_2_ 100 mg, vitamin B_6_ 100 mg, vitamin B_12_ 750 µg, vitamin K3 52.5 mg, nicotinic acid 500 mg, pantothenic acid 337.5 mg, choline chloride 5,000 mg; ^2^ > 97% SiO_2_ (Sipernat^®^ 22S, Evonic Industries, Hanau-Wolfgang, Germany).

In order to facilitate pre- or post-hepatic blood-sampling and infusion, pigs were surgically fitted with 5 differently-located, permanent indwelling catheters under general inhalation anesthesia (Isoflurane^®^, CP-Pharma, Burgdorf, Germany) at Day 27 of the trial when the animals had an average body weight of 40.5 ± 3.0 kg. Permanent Silastic^®^ catheters were manufactured from Dow Corning (Midland, TX, USA) medical-grade tubing material (1.57 mm ID and 3.18 mm OD), autoclaved and placed in the *Vena jugularis interna*, *Vena jugularis externa*, *Vena lienalis*, *Vena portae hepatis* and *Arteria carotis communis*. Catheters were tunneled to the neck and left flank, respectively, and fixed with catheter mounts/clamps (Arrow, Teleflex Medical GmbH, Kernen, Germany) on the skin. Catheters were fitted with three-way valves (Walther-CMP, Kiel, Germany) for sampling and flushed with heparinized physiological saline (1 mL sodium heparin (25.000 IE/5 mL) (Ratiopharm, Ulm, Germany); dissolved in 500 mL sterile 0.9% NaCl (B. Braun Melsungen AG, Melsungen, Germany) every 4 h and after each sampling to prevent blood coagulation. Two days between surgery and sampling day were allowed for recovery. Throughout this recovery period, half of the daily ration was fed (2 times 350 g/day).

At Day 29 of the trial, animals were further divided into 3 infusion groups: LPS pre-hepatic (LPS_portal_-CON_jugular_), LPS post-hepatic (CON_portal_-LPS_jugular_) or control (CON_portal_-CON_jugular_), illustrated in Figure 9.

On the sampling day, the time of infusion start was set as zero, and blood samples were taken at −30, 15, 30, 45, 60, 75, 90, 120, 150 and 180 min from *Vena jugularis interna, Vena portae hepatis* and *Arteria carotis communis*. Fifteen minutes prior to infusion, pigs received 700 g of feed each. LPS was infused at 7.5 µg LPS/kg BW for 1 h (*Escherichia coli*-LPS, O111:B4, Sigma-Aldrich, Taufkirchen, Germany), and 0.9% NaCl was used as the control substance. Infusion was implemented using an infusion pump (IPC-N-4, ISMATEC Laboratoriumstechnik GmbH, Wertheim, Germany) and infusion tubes with a 2.06 mm inner diameter (PharMed^®^ Ismaprene, Wertheim, Germany, ISMATEC), administered into either *Vena jugularis externa* or *Vena lienalis*. The pigs were slaughtered 195 min after infusion start.

### 4.2. Sample Analysis

For a red hemogram assessment, 1-mL blood samples were collected in EDTA tubes and analyzed immediately with an automated hematology analyzer (Celltac alpha MEK-6450, Nihon Kohden Corporation, Tokyo, Japan). Furthermore, blood samples (0.5 mL) for blood gases, electrolytes, pH, glucose and lactate were collected into blood sample syringes (SC-Sanguis Counting GmbH, Nümbrecht, Germany), and variables were assessed immediately using an automated blood gas and electrolyte analyzer (GEM Premier 4000, Werfen, Kirchheim, Germany). Anion gap and base excess were calculated using the equations detailed below.

### 4.3. Calculations

Equation used to calculate anion gap (AG) [64]:

$$AG=\left({\text{Na}}^{+}+{\text{K}}^{+}\right)-\left({\text{Cl}}^{-}+{\text{HCO}}_{3}^{-}\right)$$

Na^+^ = sodium; K^+^ = potassium; Cl^−^ = chloride; HCO_3_^−^ = bicarbonate.

Equation used to calculate base excess (BE) [65]:

$$\begin{array}{c} \text{BE}=\left(1-0.0143\;\times \text{cHb}\right)\\ \times \left[\left(0.0304\;\times p{\text{CO}}_{2}\;\times \;10^{\text{pH}-6.1}-24.26\right)+\left(9.5+1.63\;\times cHb\right)\times \left(\text{pH}-7.4\right)\right]\\ -\;0.2\;\times \text{cHb}\;\times \left(1-s{\text{O}}_{2}\right) \end{array}$$

cHb = total hemoglobin concentration; *p*CO_2_ = carbon dioxide partial pressure; *s*O_2_ = oxygen saturation.

### 4.4. Statistical Analysis

Data were evaluated by using PROC MIXED in SAS Enterprise Guide 6.1 (SAS Institute 2013, Cary, NC, USA) using a restricted maximum likelihood model (REML). Group, catheter, time and their interaction were defined as fixed factors. A “REPEATED” statement was included to account for the individual similarity at repeated measurements. The “compound symmetry” was found to be the most appropriate co-variance structure according to the corrected Akaike’s information criterion (AICC), and significant effects at different time points were further evaluated by multiple *t*-tests (“pairwise differences” (PDIFF)). Results are presented as least square means (LSMeans) and pooled standard error of means (PSEM).

## 5. Conclusions

Concluding, we could demonstrate chronic enteral DON-exposure had a definite priming effect on the pig’s organism, specifically the acid-base balance, thus aggravating the sole impact of LPS depending on the site of its entry. The involvement of the liver in this scenario was apparent as post-hepatic LPS exposure elicited a much stronger impact compared to pre-hepatic exposure in combination with mycotoxin feeding. Portal glucose uptake was significantly diminished in DON-fed animals compared to control, thus giving *in vivo* evidence to the previously-reported impairment of glucose transporter activity in *ex vivo* studies.
